# Supplementary figures and images for: 6-OHDA-induced dopaminergic neurodegeneration in Caenorhabditis elegans is promoted by the engulfment pathway and inhibited by the transthyretin-related protein TTR-33
Source: PLoS Genet. 2018 Jan 18;14(1):e1007125. doi: 10.1371/journal.pgen.1007125 (PMC5773127; doi:10.1371/journal.pgen.1007125)

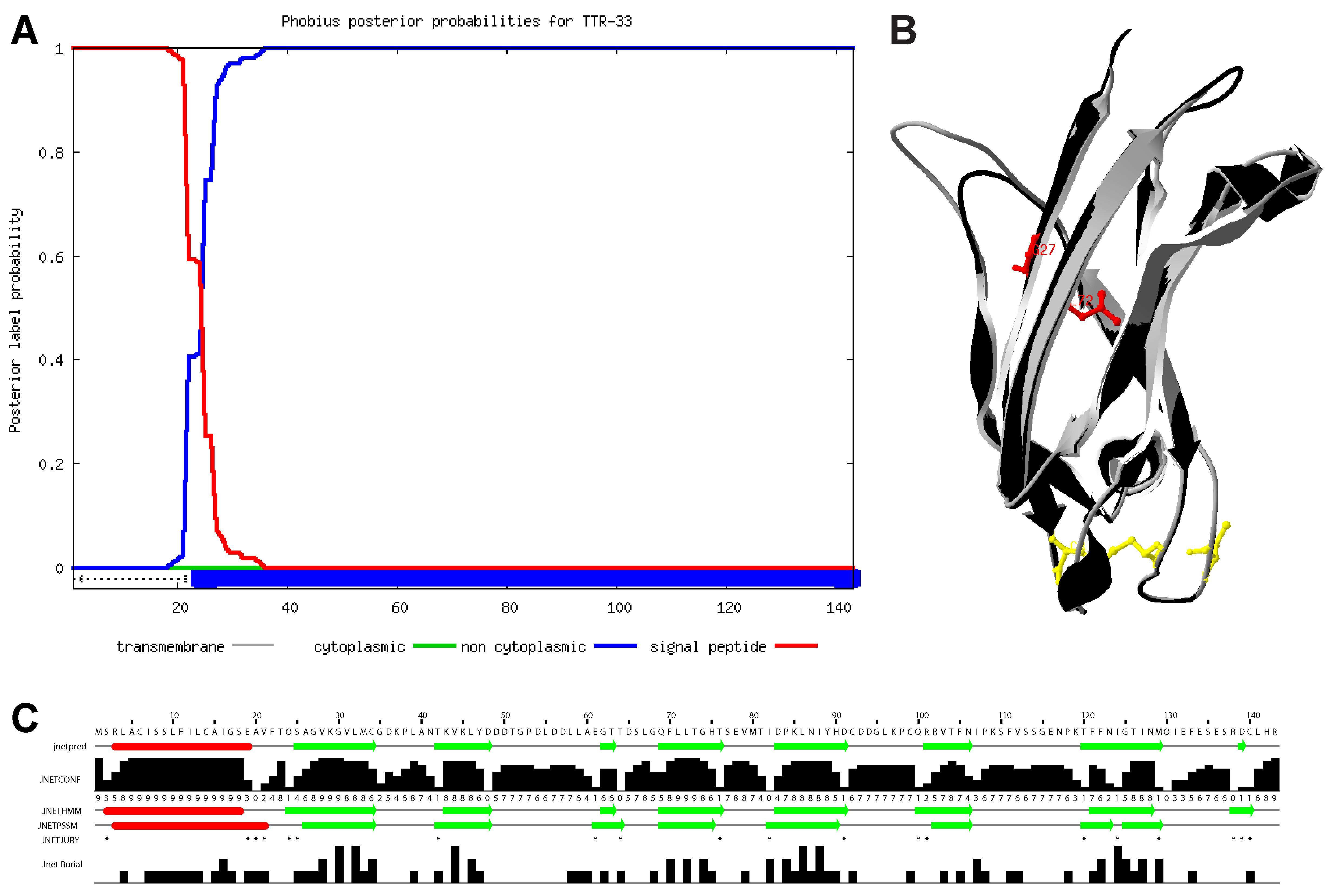

Supplement: S2 Fig — (A) TTR-33 signal peptide (in red) and non-cytoplasmic part (in blue) as determined using the Phobius webserver [63] (http://phobius.sbc.su.se/). No transmembrane domains (in grey) and no cytoplasmic parts (in green) were predicted. (B) TTR-33 secondary structure with helices (red tubes) and beta strands (green arrows) as predicted by JPred4 [67]. (C) Predicted TTR-33 structure (in grey with indicated mutated sites in red and cysteine bridges in yellow) with superimposed TTR-52 structure (in black). The small TTR-52 α-helix (top right part of structure) is likely an artefact caused by mutations that were introduced for crystallisation [25]. (TIF) [file pgen.1007125.s002.tif]

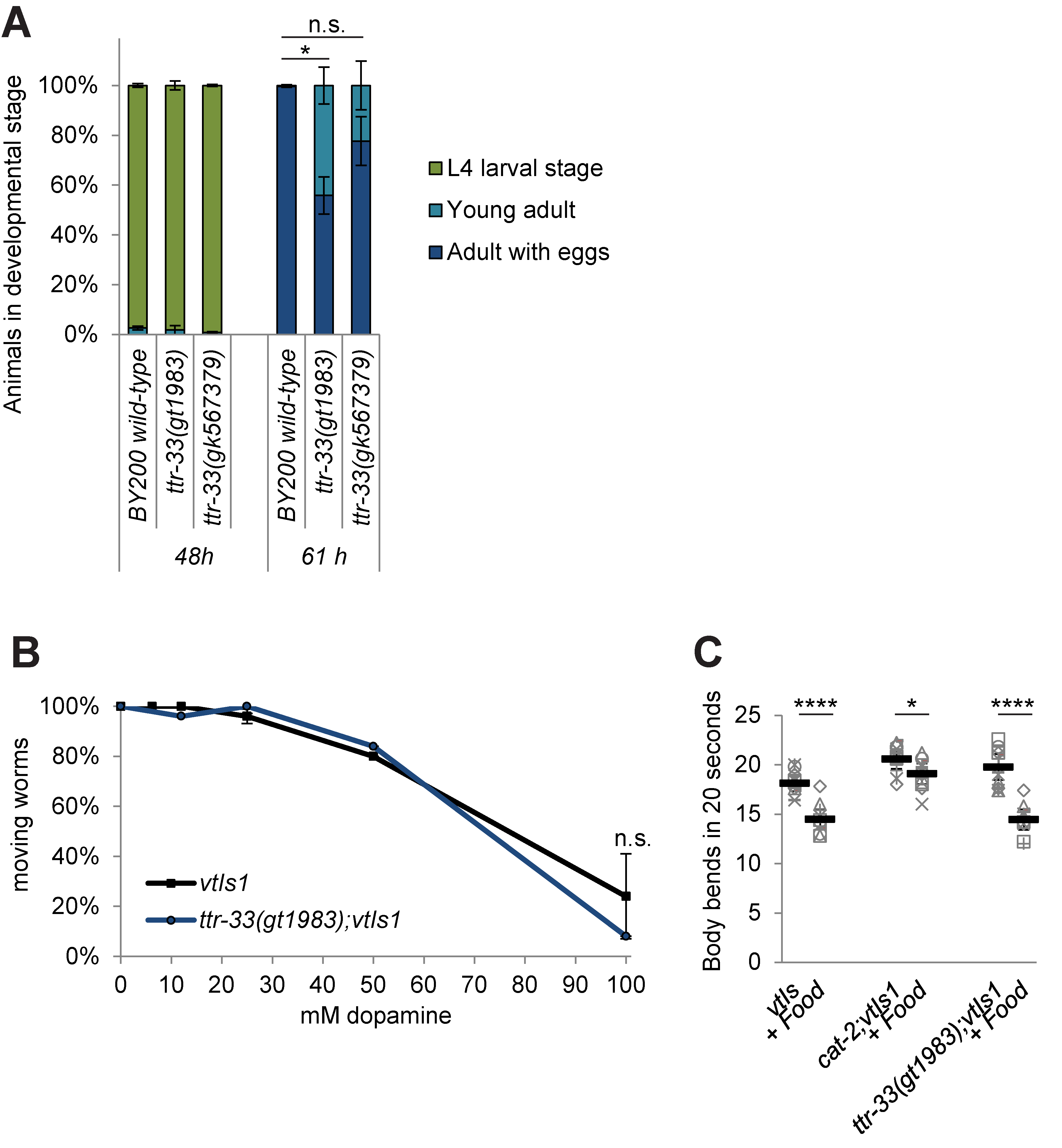

Supplement: S3 Fig — (A) Developmental stages of wild-type and ttr-33 mutant embryos 48 and 61 hours after egg-laying. Error bars = SEM of 3 biological replicates, each with 40–230 animals per strain. Total number of animals n = 405–515 (*p<0.05, n.s. p>0.05; G-Test). (B) Dopamine paralysis assay: Ability of young adult animals to move on plates with indicated concentrations of dopamine. Error bars = StDev of 2 technical replicates, each with 25 animals per strain and condition. Total number of animals per condition n = 50. (n.s. p>0.05; two-tailed t-test comparing wild-type and mutant animal data at 100 mM dopamine). (C) Basal slowing response: Ability of young adult animals to slow down on a lawn of bacteria. The tyrosine hydroxylase mutant cat-2 (abnormal catecholamine distribution) is deficient of dopamine synthesis. The values for each animal are depicted with grey symbols and the average across animals is indicated with a black bar. Error bars = SEM of 2 biological replicates, each with 6 animals per strain and state. Total number of animals per condition n = 12 (****p<0.000001, *p<0.05; two-tailed t-test). (TIF) [file pgen.1007125.s003.tif]

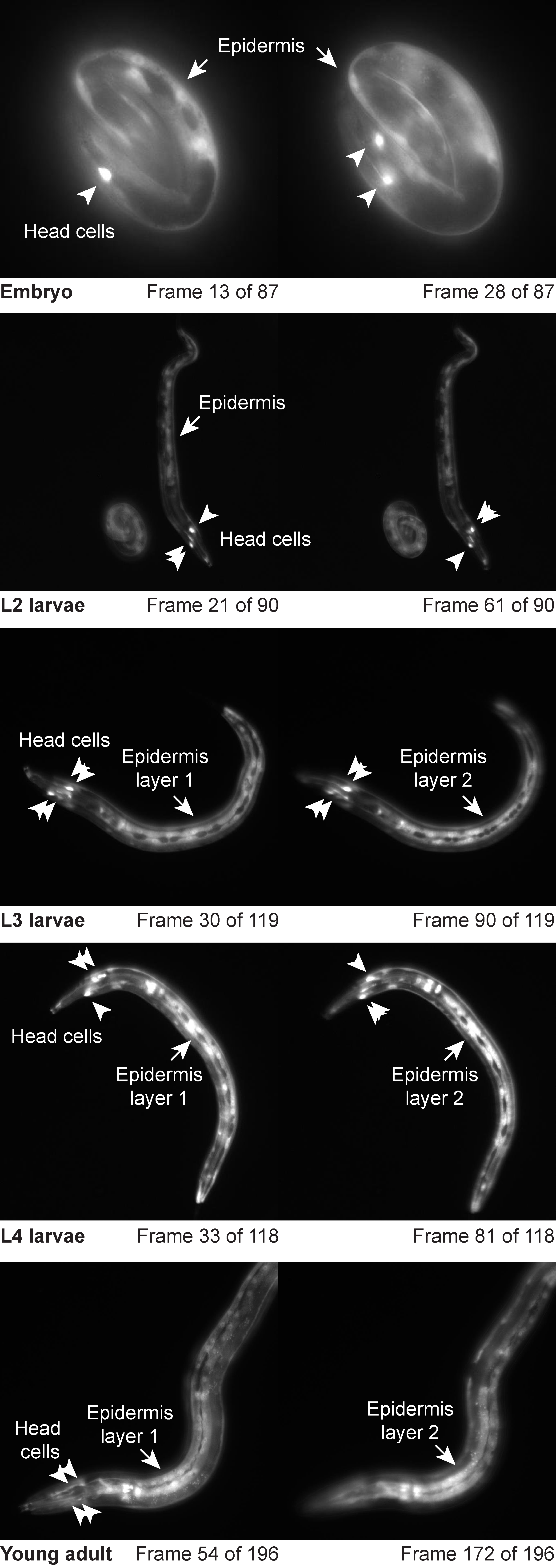

Supplement: S6 Fig — Head cells (likely arcade cells) are labelled with arrowheads, epidermal staining with arrows. (TIF) [file pgen.1007125.s006.tif]

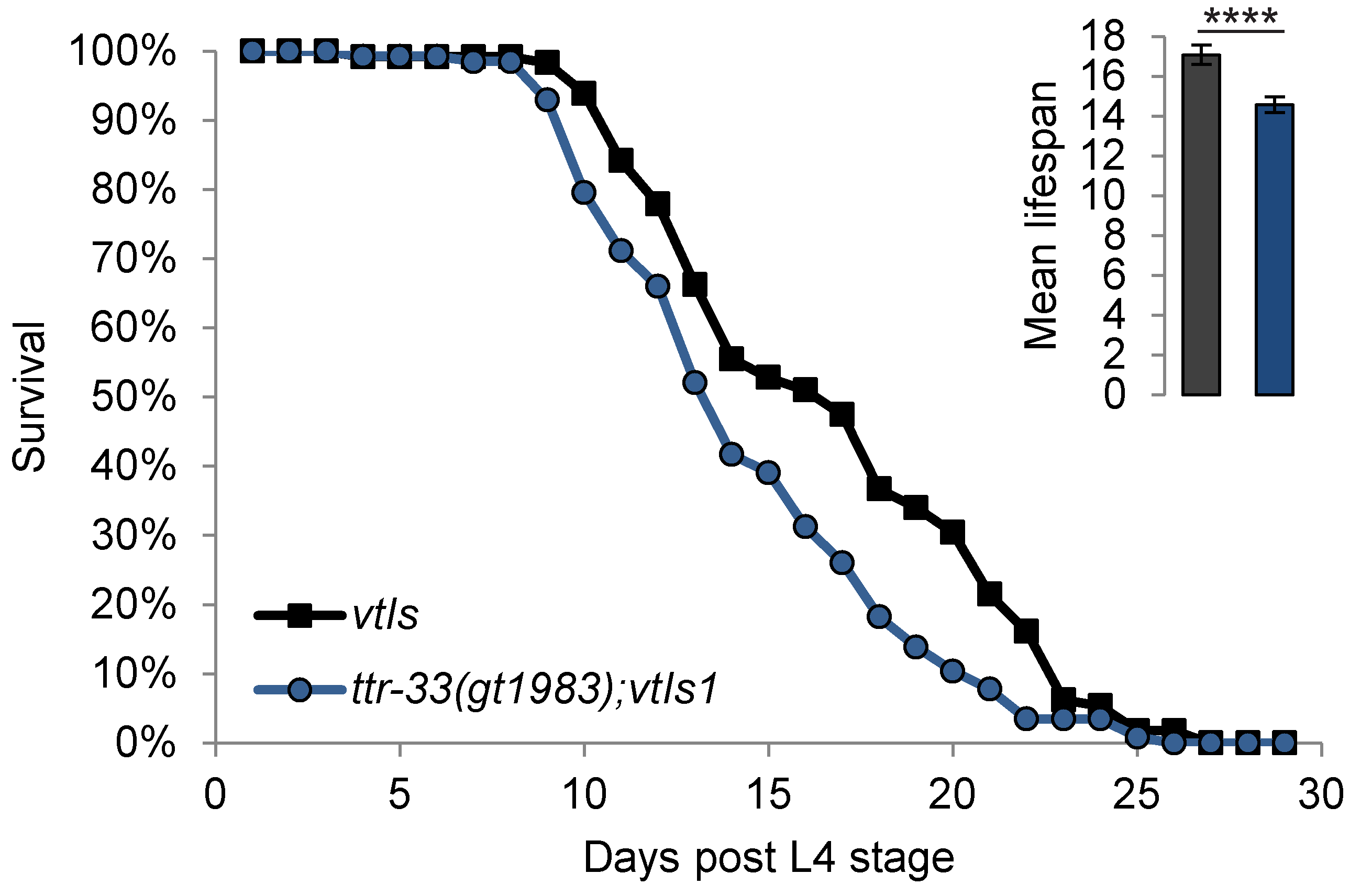

Supplement: S7 Fig — Lifespan data for second biological replicate including 85–110 animals per strain. The inset shows the mean lifespan with the error bars depicting the standard error (****Bonferroni p≤0.0001; Log-Rank Test). (TIF) [file pgen.1007125.s007.tif]

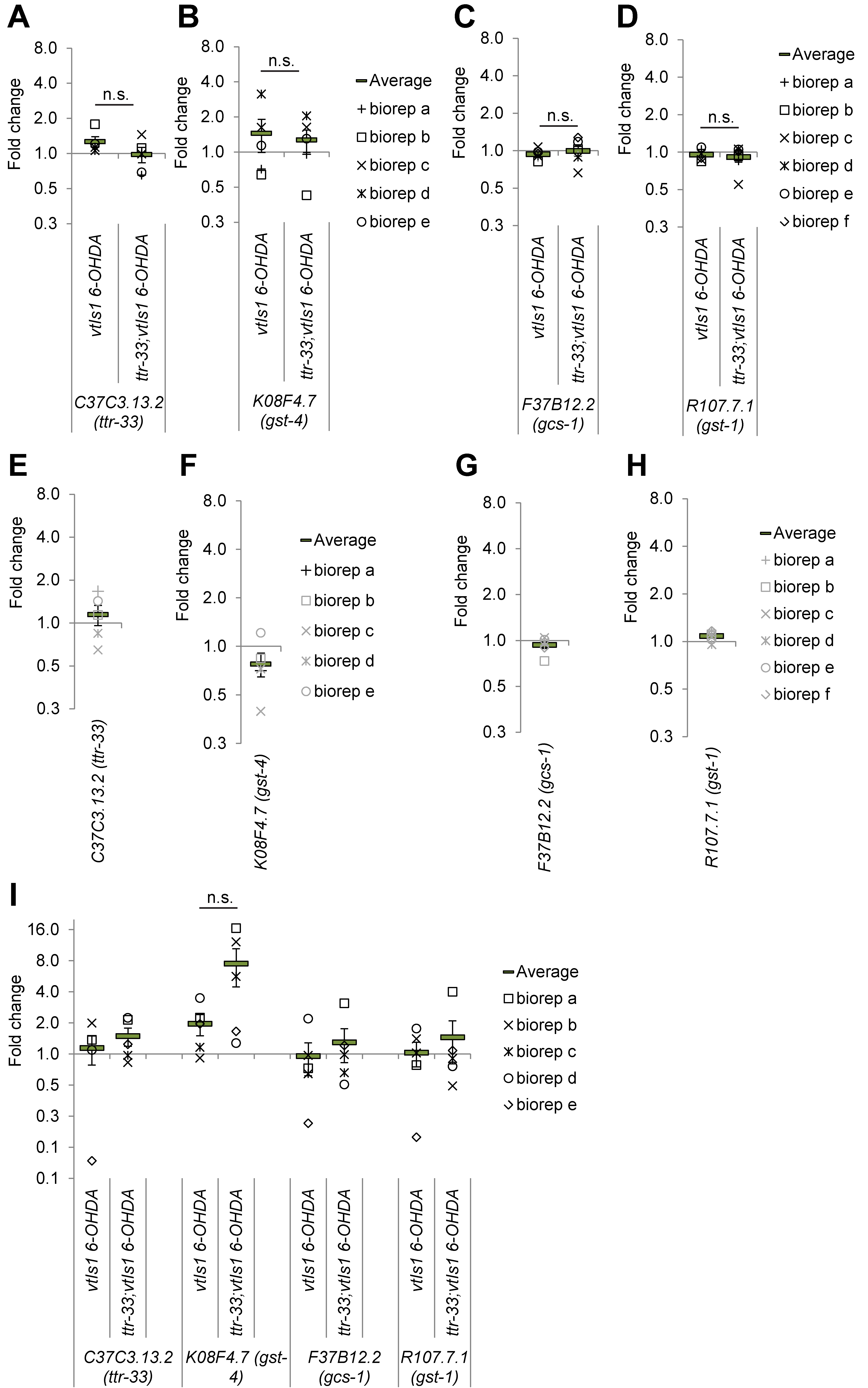

Supplement: S8 Fig — (A) ttr-33, (B) gst-4, (C) gcs-1 and (D) gst-1 mRNA levels in wild-type and ttr-33 mutant L1 stage larvae after treatment with 10 mM 6-OHDA. (E) ttr-33, (F) gst-4, (G) gcs-1 and (H) gst-1 mRNA levels in ttr-33 mutants at L1 stage as compared to wild-type larvae under control conditions (no 6-OHDA exposure). (I) ttr-33, gst-4, gcn-1 and gst-1 mRNA levels in wild-type and ttr-33 mutant L1 stage larvae after treatment with 25 mM paraquat. (A)-(I) The data are normalised to the control gene Y45F10D.4 [61,62]. The average and the respective values for the biological replicates (biorep a-f) are indicated. Error bars = SEM of 5–6 biological replicates (n.s. p>0.05). (TIF) [file pgen.1007125.s008.tif]

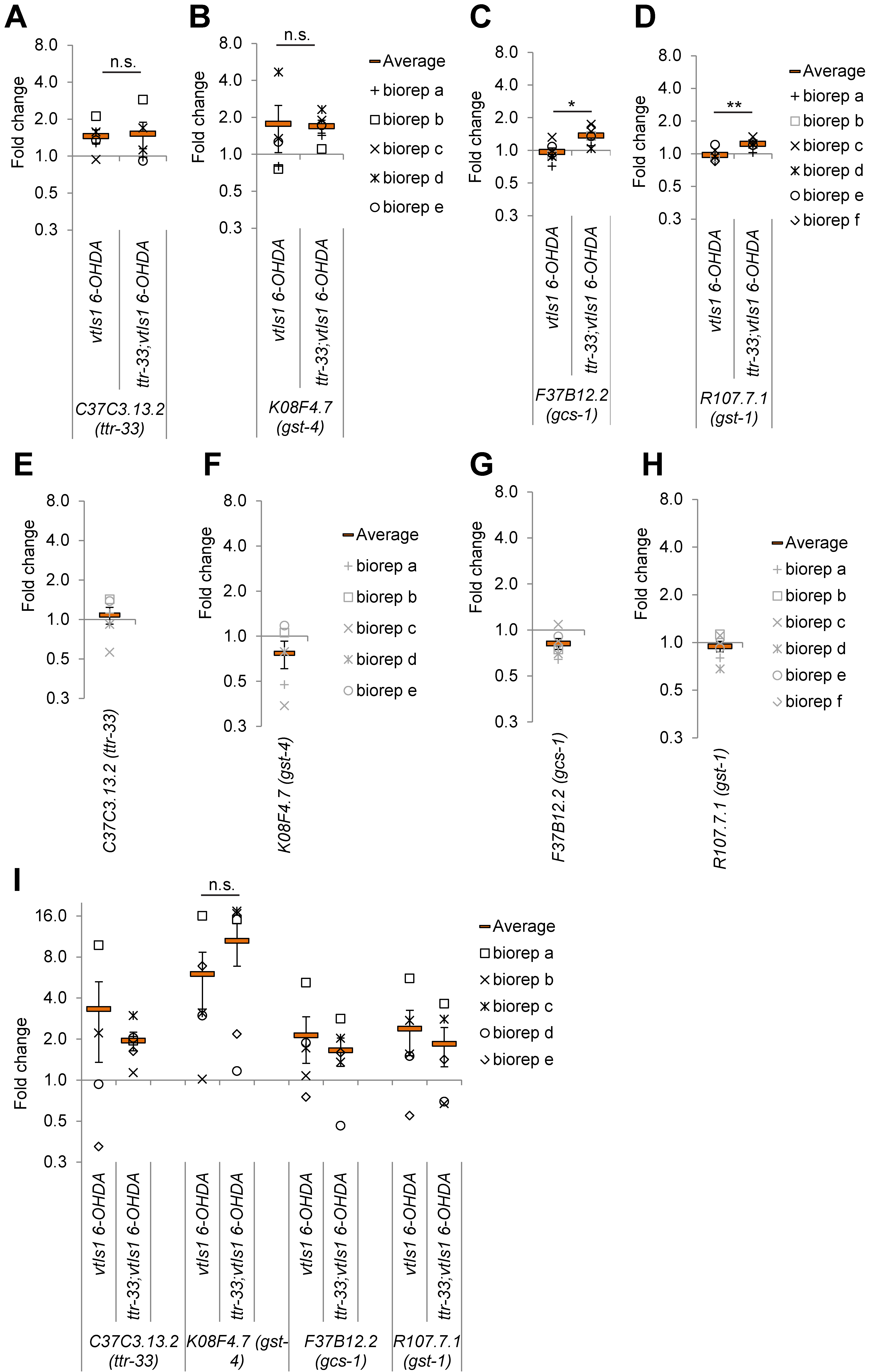

Supplement: S9 Fig — (A) ttr-33, (B) gst-4, (C) gcs-1 and (D) gst-1 mRNA levels in wild-type and ttr-33 mutant L1 stage larvae after treatment with 10 mM 6-OHDA. (E) ttr-33, (F) gst-4, (G) gcs-1 and (H) gst-1 mRNA levels in ttr-33 mutant at L1 stage as compared to wild-type larvae under control conditions (no 6-OHDA exposure). (I) ttr-33, gst-4, gcn-1 and gst-1 mRNA levels in wild-type and ttr-33 mutant L1 stage larvae after treatment with 25 mM paraquat. (A)-(I) The data are normalised to the control gene pmp-3 (C54G10.3b) [61,62]. The average and the respective values for 5–6 biological replicates (biorep a-f) are indicated. Error bars = SEM of 5–6 biological replicates (**p<0.01, *p<0.05; n.s. p>0.05; two-tailed t-test). (TIF) [file pgen.1007125.s009.tif]

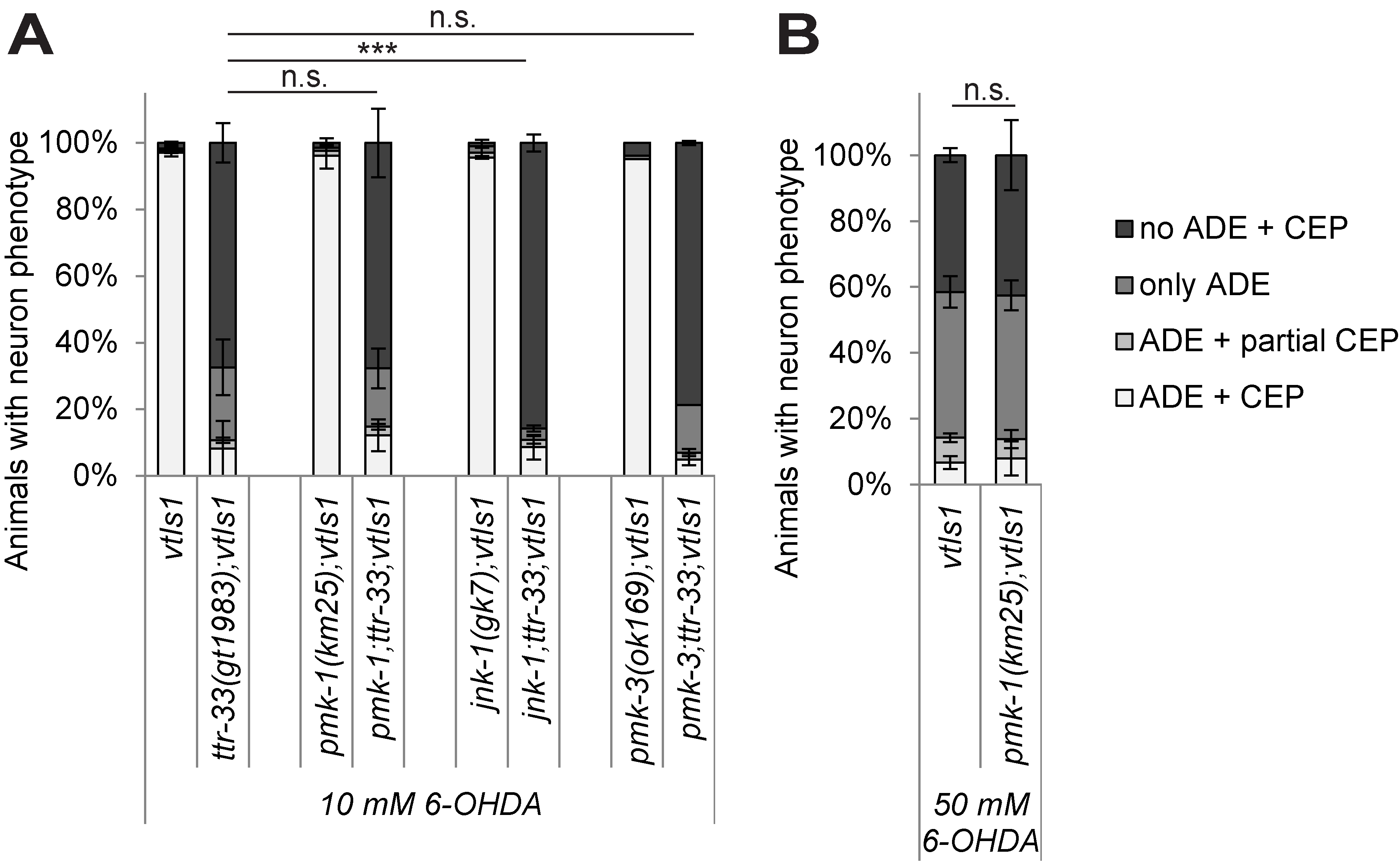

Supplement: S10 Fig — (A) Effect of p38 and JNK stress response pathway mutations on dopaminergic neurodegeneration after treatment with 10 mM 6-OHDA. Error bars = SEM of 2–3 biological replicates, each with 100–115 animals per strain and concentration. Total number of animals per condition n = 100–315 (***p<0.001, n.s. p>0.05; G-Test). (B) Effect of p38 and JNK stress response pathway mutations on dopaminergic neurodegeneration after treatment with 50 mM 6-OHDA. Error bars = SEM of 3 biological replicates, each with 100–110 animals per strain. Total number of animals per strain n = 305–320 (n.s. p>0.05; G-Test). (TIF) [file pgen.1007125.s010.tif]

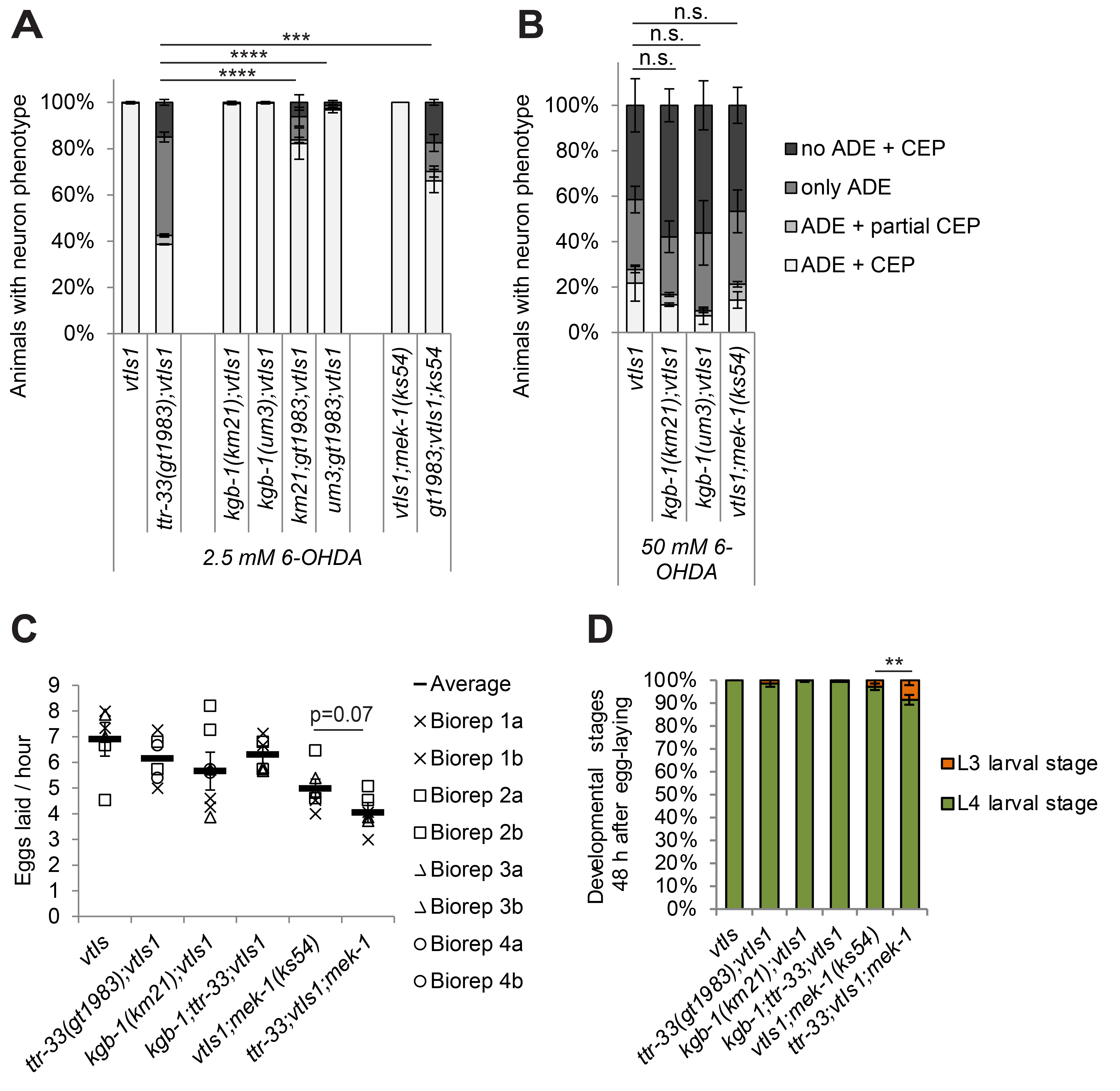

Supplement: S11 Fig — (A) Effect of kgb-1 MAP kinase pathway mutations on dopaminergic neurodegeneration after treatment with 2.5 mM 6-OHDA. Error bars = SEM of 2–3 biological replicates, each with 95–120 animals per strain. Total number of animals per strain n = 200–340 (****p<0.0001, ***p<0.001; G-Test). (B) Effect of kgb-1 MAP kinase pathway mutations on dopaminergic neurodegeneration after treatment with 50 mM 6-OHDA. Error bars = SEM of 3 biological replicates, each with 100–125 animals per strain. Total number of animals per strain n = 205–335 (n.s. p>0.05; G-Test). (C) Number of laid eggs per animal per hour. Error bars = SEM of 3–4 biological replicates, each with 20 animals per strain. Total number of animals n = 60–80 (two-tailed t-test). (D) Developmental stages of wild-type and mutant embryos 48 hours after egg-laying. Error bars = SEM of 3 biological replicates, each with 40–125 animals per strain. Total number of animals n = 150–350 (**p<0.01; two-tailed t-test). (TIF) [file pgen.1007125.s011.tif]

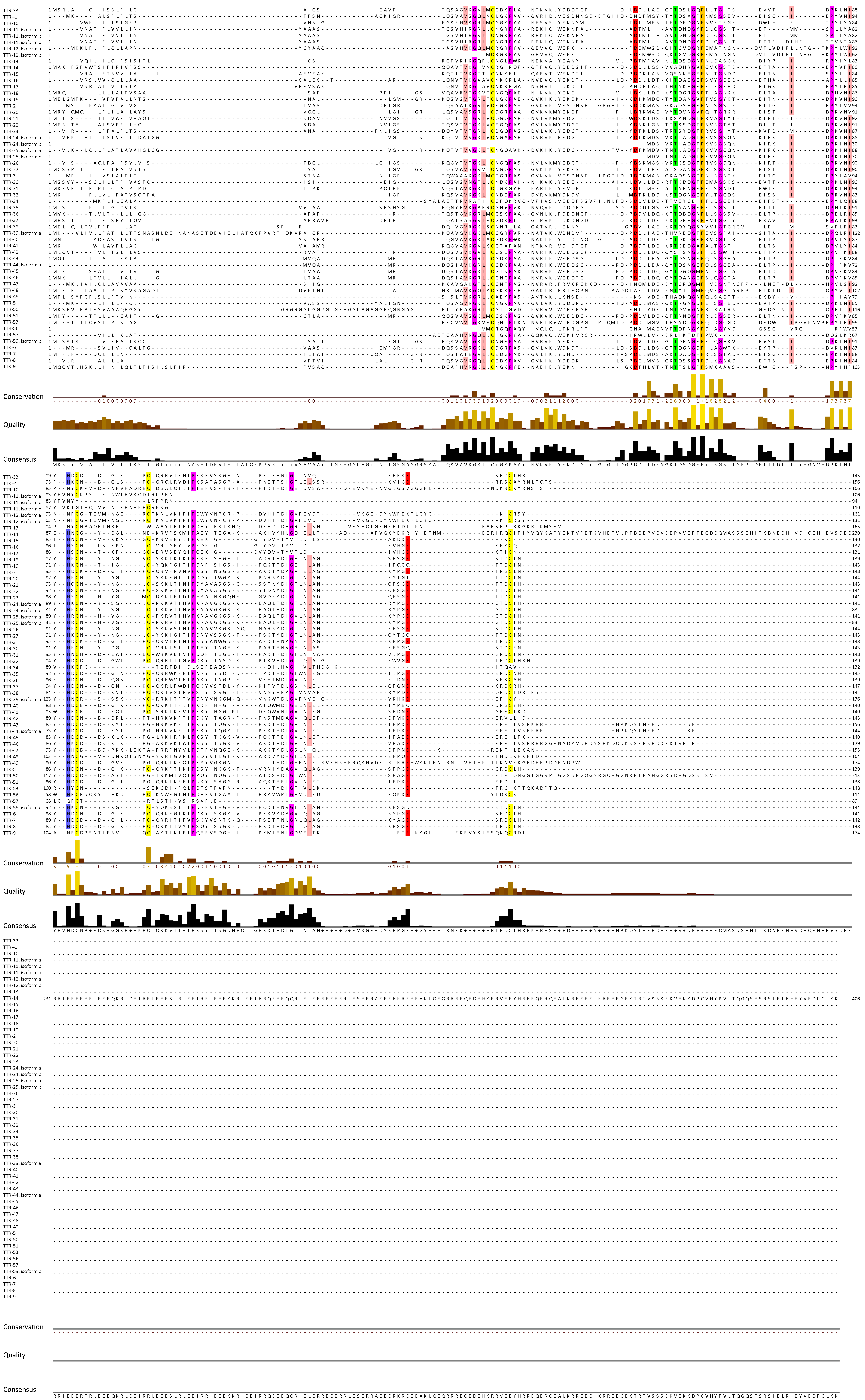

Supplement: S12 Fig — Amino acids above an identity threshold of 69 are coloured according to their physicochemical properties using the Zappo colour code. Conservation score, quality and consensus are each shown as a bar graph in the bottom of the alignment. (TIF) [file pgen.1007125.s012.tif]
